# Stress-Derived Corticotropin Releasing Factor Breaches Epithelial Endotoxin Tolerance

**DOI:** 10.1371/journal.pone.0065760

**Published:** 2013-06-19

**Authors:** Yong Yu, Zhi-Qiang Liu, Xiao-Yu Liu, Li Yang, Xiao-Rui Geng, Gui Yang, Zhi-Gang Liu, Peng-Yuan Zheng, Ping-Chang Yang

**Affiliations:** 1 Department of Gastroenterology, the Second Hospital, Zhengzhou University, Zhengzhou, China; 2 Allergy & Immunology Institute, Shenzhen University School of Medicine, Shenzhen, China; 3 Longgang Central Hospital, ENT Hospital and Shenzhen ENT Institute, Shenzhen, China; Rush University Medical Center, United States of America

## Abstract

**Background and aims:**

Loss of the endotoxin tolerance of intestinal epithelium contributes to a number of intestinal diseases. The etiology is not clear. Psychological stress is proposed to compromise the intestinal barrier function. The present study aims to elucidate the role of the stress-derived corticotropin releasing factor (CRF) in breaching the established intestinal epithelial endotoxin tolerance.

**Methods:**

Epithelial cells of HT-29, T84 and MDCK were exposed to lipopolysaccharide to induce the endotoxin tolerance; the cells were then stimulated with CRF. The epithelial barrier function was determined using as indicators of the endotoxin tolerant status. A water-avoid stress mouse model was employed to test the role of CRF in breaching the established endotoxin tolerance in the intestine.

**Results:**

The established endotoxin tolerance in the epithelial cell monolayers was broken down by a sequent exposure to CRF and LPS manifesting a marked drop of the transepithelial resistance (TER) and an increase in the permeability to a macromolecular tracer, horseradish peroxidase (HRP). The exposure to CRF also increased the expression of Cldn2 in the epithelial cells, which could be mimicked by over expression of TLR4 in epithelial cells. Over expression of Cldn2 resulted in low TER in epithelial monolayers and high permeability to HRP. After treating mice with the 10-day chronic stress, the intestinal epithelial barrier function was markedly compromised, which could be prevented by blocking either CRF, or TLR4, or Cldn2.

**Conclusions:**

Psychological stress-derived CRF can breach the established endotoxin tolerance in the intestinal mucosa.

## Introduction

The intestinal epithelial endotoxin tolerance is defined as a reduced capacity of the host (*in vivo*) to respond to lipopolysaccharide (LPS) activation following a first exposure to this stimulus [1]. Naïve intestinal epithelial cells do not have such a tolerant mechanism; it can be established once exposure to LPS [2]. The discovery of Toll like receptor (TLR)4 and myeloid differentiation (MD)-2 has better described the mechanism of endotoxin tolerance [1], [3]. Yet, mechanisms of breaching the established endotoxin tolerance are to be further understood. One of the major consequences of the disturbance of intestinal epithelial endotoxin tolerance is to induce the epithelial barrier dysfunction, which is involved in the pathogenesis of a number of intestinal disorders, such as inflammatory bowel disease (IBD) [4], the latter affects about 0.1%–0.5% population in the world [5], [6].

Published data indicate that psychological stress plays an important role in the intestinal epithelial barrier dysfunction in animal models [7]–[9], in which the intestinal endotoxin tolerance is supposed to have been established before the stress. The corticotropin releasing factor (CRF) is an important mediator in the stress-induced pathophysiological changes in the body [7]–[10]. Eosinophils in the intestine can be a source of the CRF, which can be released during psychological stress [10], [11]. Further studies revealed that blocking the receptors of CRF could abrogate the effect of stress on the intestinal epithelial barrier function [9], [12]. Mast cells were found involved in the stress induced epithelial barrier dysfunction as reported by several research groups [7], [13]–[15]; others also indicate that additional pathways other than mast cell activation are involved in stress-induced intestinal dysfunction [16]. To date, the underlying mechanisms by which psychological stress induced intestinal epithelial barrier dysfunction are to be further investigated.

We and others [9], [17] observed that the intestinal epithelial permeability was markedly increased in the experimental animals after treating with psychological stress,; the fact implicates the tight junction complex is disturbed during the stress. The tight junction complexes are composed of several proteins including occludins and claudins. The latter is a protein family with more than 20 members [18]. Studies indicate that not all the tight junction associated proteins contribute to maintaining the integration of tight junction; some of them, such as claudin 2 (Cldn2), can compromise the barrier function [19]. The factors inducing Cldn2 expression in intestinal epithelial cells are not clear.

By integrating the information of psychological stress-induced intestinal barrier dysfunction and the concept of intestinal endotoxin tolerance, we hypothesized that the psychological stress-derived CRF might be involved in breaching the established intestinal endotoxin tolerance that further induced the barrier dysfunction. With an intestinal epithelial cell monolayer model and a psychological stress mouse model, we observed that exposure to CRF increased the expression of TLR4 in the epithelial cell line, HT-29, T84 and MDCK cells, that breached the established tolerance to LPS. Further observation indicated that CRF increased the expression of Cldn2 in intestinal epithelial cells, which contributed to the intestinal epithelial barrier dysfunction.

## Materials and Methods

### Quantitative real-time RT-PCR (qRT-PCR)

Total RNA was extracted from cells or mouse intestinal epithelial tissue using Trizol Reagents. The template cDNA was reverse transcribed from 1 µg of RNA using a cDNA synthesis kit. SYBR green-based qRT-PCR was performed with a Bio-Rad MiniOpticon™ Real-Time PCR Detection System. Expression of target genes was normalized to β-actin mRNA levels. Primer sequences for TLR (2–4) and tight junction associated proteins using in this study were presented in Table S1.

### Determining the epithelial monolayer barrier function

The epithelial cell culture was described in supplemental materials. The transepithelial resistance (TER) and permeability to a macromolecular tracer, horseradish peroxidase (HRP) or dextran, were recorded using as the indicators of the epithelial barrier function following our established procedures [10], [20], or measured at 492/520 nm (excitation/emission) with a pectraMax Gemini XS system (Conquer Scientific, San Diego, CA).

### Knockdown of genes of TLR4 and Cldn2 in epithelial cells by gene silencing

HT-29 cells were transduced with a commercial lentiviral shTLR4 kit, or a lentiviral shCldn2 kit; using control shRNA as a control. The effect of gene knockdown was presented in Fig.S1.

### Over-expression of TLR4 in epithelial cells

HT-29 cells were transfected with commercial plasmid of pTLR4 or cpTLR4 following the manufacturer's instruction. The results of pTLR4 transfection were depicted in Fig.S2.

### Statistical analysis

All data were expressed as mean ± SD of three or more individual experiments. Statistical comparisons among groups were performed with one-way analysis of variance (ANOVA) and the Student *t* test. p value<0.05 was considered significant.

Other experimental procedures were presented in the supplemental materials.

## Results

### CRF induces the expression of TLR4 in intestinal epithelial cells

**Figure 1:**
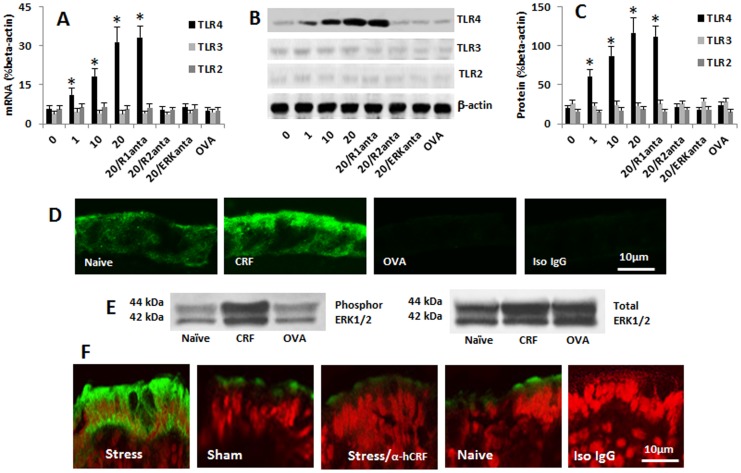
CRF induces TLR4 expression in HT-29 cells. Confluent HT-29 cells were stimulated by the addition of CRF in the culture for 24 h. The expression of TLR2, TLR3 and TLR4 in HT29 cells was evaluated by qRT-PCR and Western blotting. The bars indicate the levels of TLR mRNA (A; by qRT-PCR) and proteins (B) in HT-29 cells. Panel C is the integrated density of the immune blots in panel B. R1 (R2, ERK) anta: Cells were treated with the CRF R1 antagonist (astressin_2_B; 300 nM), CRF R2 antagonist (antalarmin, 300 nM), or ERK1/2 inhibitor (PD98059, 50 µM) before exposure to CRF. The data in bar graph were presented as mean ± SD. *, p<0.01, compared with dose “0” group (the naïve group). OVA: Cells were treated with OVA (100 ng/ml; an irrelevant protein control) in the culture. D, the representative confocal images indicate the staining of TLR4 (in green) in HT-29 monolayers; the treatment was annotated in each image. Con: Isotype control. E, the immune blots show the levels of phosphor ERK1/2 and total ERK1/2 in the cell extracts of HT-29 cells. The data represent 5 separate experiments. F, the representative confocal images show the staining of TLR4 (in green) in the colon epithelial cells of fetal mice. Stress: The pregnant mice were treated with water-avoid stress daily for 10 days. α-hCRF: Mice were treated with α-helical CRF (an antagonist of CRF; 5 ng/mouse, i.p., 30 min before each stress session). Sham: Mice were treated with sham stress. Naïve: Naïve mice. Iso IgG: The section was stained with isotype IgG using as a staining control. Each group consisted of 6 mice.

To clarify if psychological stress modifies the ability of intestinal epithelial cells in recognizing the stimulation of endotoxin, we treated human colon epithelial cell line, HT-29 cells, with five of the psychological stress-related molecules including CRF, corticosterone, norepinephrine, prolactin and adrenocorticotropic hormone [21]–[23]. The expression of TLR2, TLR3 and TLR4 in HT-29 cells was assessed. The results showed that the expression of TLR2, TLR3 and TLR4 was detected in naive HT-29 cells. The expressions of TLR4 in HT-29 cells were significantly increased after the exposure to CRF in a dose-dependent manner, but did not increase after exposure to other four molecules (Fig. 1A–B; Table S2). The expression of TLR2 and TLR3 was not changed much (Fig. 1A–B; Table S2). To understand the effect of CRF on the expression of TLR4 by HT-29 cells was mediated by CRF receptor (R)1 or CRF R2, the HT-29 cells were treated with either CRF R1 or CRF R2 antagonist 30 min before the exposure to CRF; the effect of CRF on the TLR4 expression in HT-29 cells was blocked by the CRF R2 antagonist, but not R1 antagonist (Fig. 1A–C). The data of CRF-increasing the TLR4 expression on HT-29 cells were confirmed by immune staining (Fig. 1D). The results indicate that exposure to CRF can increase the expression of TLR4 in HT-29 cells via activation of CRF R2.

It is reported that the activation of ERK1/2 is the downstream of signal transduction pathway of CRF induced bio-activities [24]. To elucidate if activation of ERK1/2 was also involved in the CRF-induced expression of TLR4 in HT-29 cells, the HT-29 cells were pretreated with the antagonist (PD98059) of ERK1/2, then stimulated by CRF; the expression of TLR4 was abolished (Fig. 1A–C). The data indicate that the activation of ERK1/2 is also involved in the CRF-induced TLR4 expression in HT-29 cells; the latter was further confirmed by Western blotting data, in which the total ERK1/2 and phosphor ERK1/2 levels in the HT-29 cells were in agreement with the levels of TLR4 (Fig. 1E).

To clarify if the stress-derived CRF could increase the expression of TLR4 in the intestinal epithelial cells *in vivo*, we treated pregnant mice with water avoid stress or sham stress daily from day 8 to day 17 during the gestation. The mice were sacrificed on day 18 before the delivery. As shown by immunohistochemistry, the expression of TLR4 was detected in the mice treated with stress, which was significantly stronger than that in naïve mice or the mice treated with sham stress. The stress-induced TLR4 expression was abolished in mice pretreated with CRF antagonists (Fig. 1F).

### Exposure to CRF breaches the established endotoxin tolerance in intestinal epithelial cells

**Figure 2:**
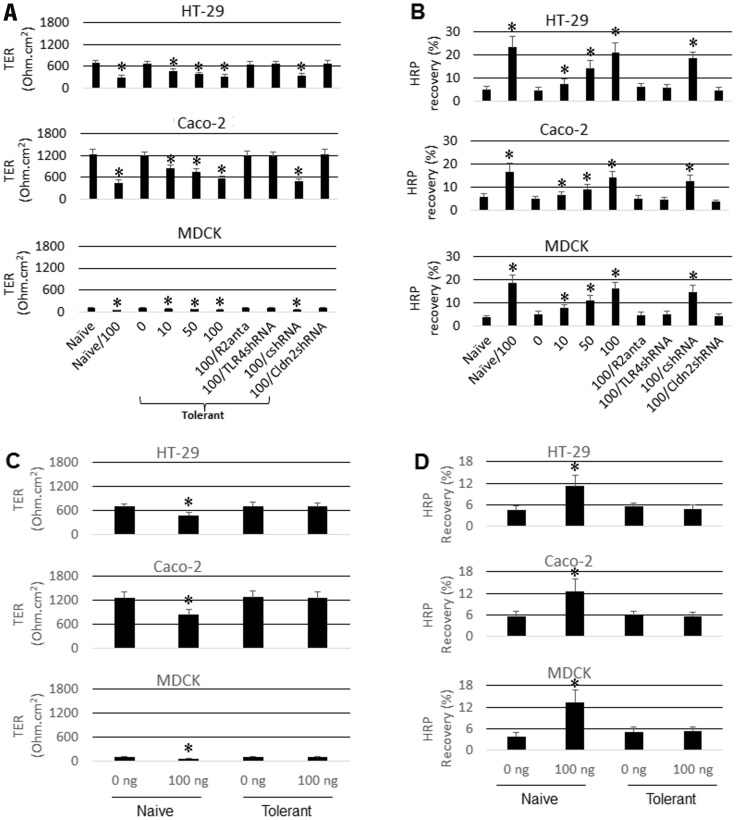
Exposure to CRF breaches the established endotoxin tolerance. Epithelial cells were cultured into monolayers, treated with or without LPS (100 ng/ml) overnight; washed with pre-warmed fresh culture medium; then, CRF (A and B) or LPS (C and D) was added to the culture at the indicated doses and cultured for 48 h. The TER was recorded at the 46^th^ h later; the HRP flux was carried out between the 46^th^ h to 48^th^ h. A and C, the bars indicate the values of TER recorded from the monolayers. B and D, the bars indicate the contents of HRP (detected in basal chambers of transwells that passed through the monolayers from apical chambers). The annotations on X axis indicate the treatment of each group. The numbers on the X axis indicate the amounts (ng/ml) of CRF (A, C) or LPS (C, D) added to the culture medium. Naïve: Naïve confluent monolayers. Tolerant: The epithelial monolayers had been treated with LPS overnight. R2anta: The monolayers were pretreated with CRF R2 antagonist, astressin_2_B at a dose of 300 nM. TLR4shRNA: TLR4-deficient monolayers (by shRNA of TLR4). csh: The monolayers were treated with control shRNA. Cldn2shRNA: Cldn2-deficient monolayers (treated by shRNA of Cldn2). The data represent 3 separate experiments.

Intestinal epithelial cells naturally developed tolerance to the commensal microbes in the intestinal tract. Such tolerance may be breached under pathological circumstances, such as in the intestine of patients with inflammatory bowel disease (IBD) [25]. It is observed that exposure to microbial products, such as LPS, is associated with intestinal chronic inflammation [26]; LPS is the ligand of TLR4; we postulated that the increase in TLR4 expression in the intestinal epithelial cells might exaggerate the responses of intestinal epithelial cells to the stimulus of LPS and thus breached the established endotoxin tolerance. To test the inference, following published procedures [27], we firstly treated the epithelial cell monolayers (including HT-29, T84 and MDCK) with LPS (100 ng/ml, overnight) to establish the endotoxin tolerance (the LPS dose “0” group of Fig. 2). The tolerant epithelial monolayers were re-exposed to LPS in the presence or absence of CRF in the culture for 48 h. The results showed that the endotoxin tolerance of the epithelial monolayer was breached by the presence of CRF as shown by the increase in the permeability to a protein tracer, HRP, and the decrease in TER. To confirm the role of CRF in the breaching the established endotoxin tolerance, some epithelial monolayers were pretreated with CRF-R2 antagonist, astressin_2_B; the results showed that the epithelial barrier function was not affected by the exposure to LPS. Similar results were observed in TLR4-deficient epithelial cell monolayers (Fig.S1A) after stimulated by CRF (Fig. 2). The results indicate that the established endotoxin tolerance in epithelial cells can be breached by the presence of CRF via increasing the expression of TLR4 in epithelial cells. In other words, the silencing TLR4 gene can “avoid” the decrease in TER induced by CRF. With the barrier function as indicators, we also checked the response to LPS-stimulation in the tolerant epithelial cells. The results showed that the barrier function was not significantly altered in the tolerant epithelial monolayers, but markedly compromised in naïve epithelial monolayers (Fig. 2C–D).

### Exposure to CRF-increased Cldn2 expression is responsible for the barrier dysfunction in HT-29 monolayers

**Figure 3:**
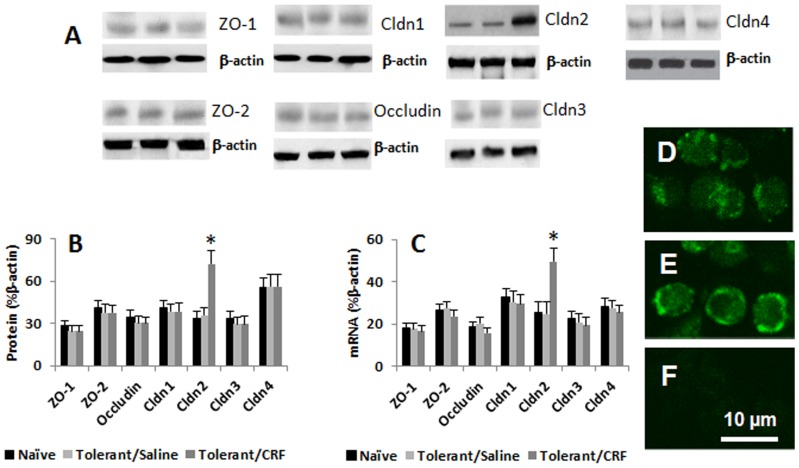
CRF induces the expression of Cldn2 in epithelial cells. The tolerant HT-29 cells were treated with saline or CRF for 24 h; then all the cells were exposed to LPS (100 ng/ml) for another 24 h; the cell extracts were examined by qRT-PCR and Western blotting. A, the immune blots indicate the tight junction associated protein levels in gut epithelial cells. B, the bars indicate the summarized integrated density of the immune blots of panel A. C, the bars indicate the levels of mRNA of the 7 tight junction proteins in HT-29 cells. D–E, representative confocal images show the positive staining of Cldn2 on naïve (D) and CRF-treated (E) HT-29 cells. F is a negative staining control. The data of B and C were presented as mean ± SD. *, p<0.01, compared with naïve groups. The data represent 5 separate experiments.

**Figure 4:**
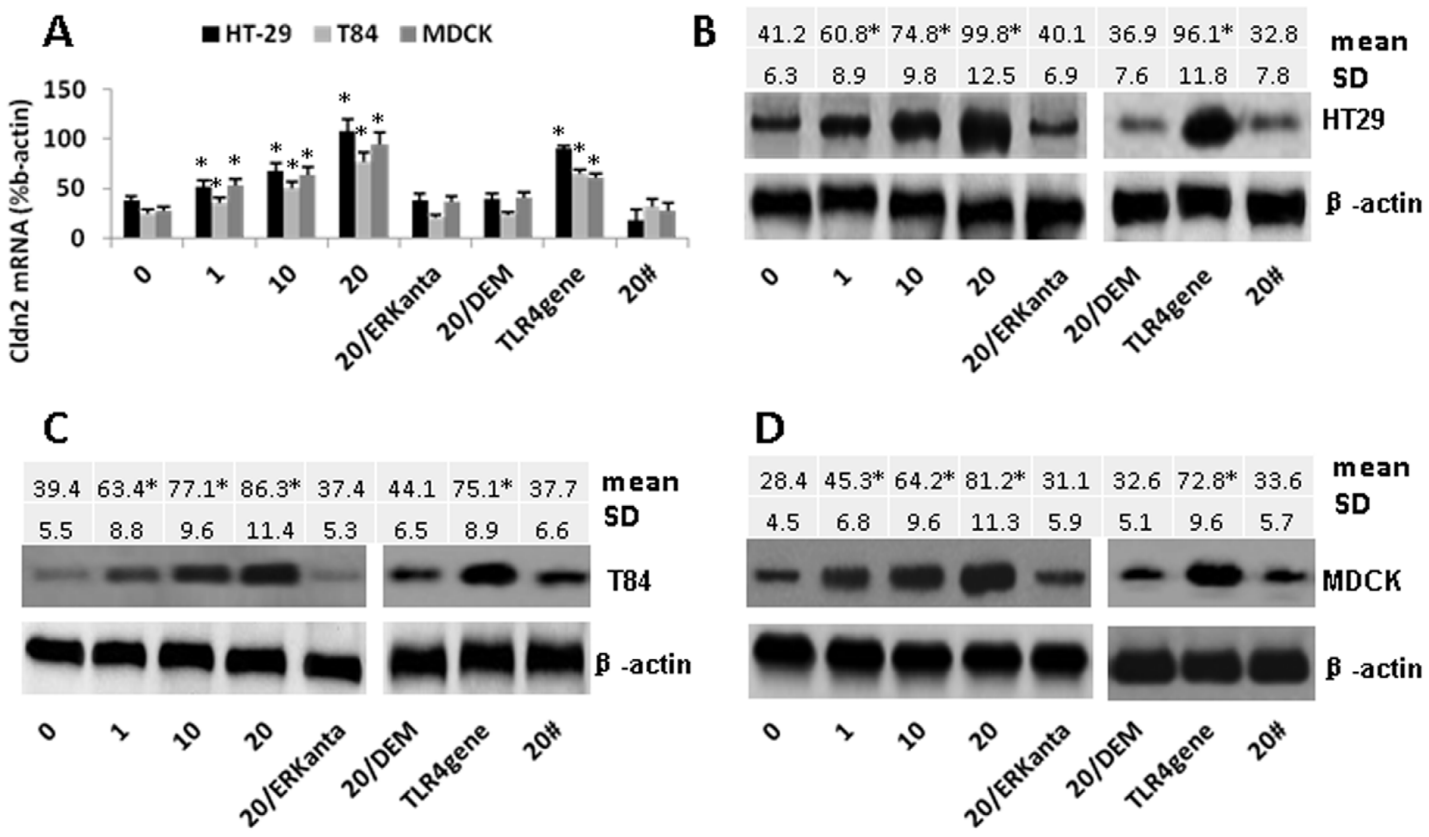
Cldn2 levels in epithelial cells. HT-29 cells, T84 cells and MDCK cells were exposed to CRF (0–20 ng/ml; indicated on the X axis) for 24 h. The bars indicate the levels of Cldn2 mRNA (A) and protein (B–D) in epithelial cell extracts. ERKanta: Cells were pretreated with ERK1/2 inhibitor (PD98059, 50 µM). DEM: Cells were pretreated with NF-κB inhibitor, diethyl maleate (DEM; 100 µM) for 30 min, and then treated with CRF. #: Cells were not treated with LPS. TLR4 gene (or TLR4con): Cells were transfected with TLR4 gene plasmid (or control plasmid); these cells were not treated with CRF. The immune blots of panel B–D indicate the levels of Cldn2 in gut epithelial cell extracts. The table above the immune blots indicate the integrated density of the immune blots. The data were presented as mean ± SD. *, p<0.01, compared with the “0” group. The data represent 5 separate experiments.

Published data indicate that Cldn2 is over expressed in IBD intestinal epithelial cells [28]; Cldn2 has the property to compromise the barrier function [19]. To further understand the underlying mechanism by which exposure to CRF breached the established endotoxin tolerance as shown by Fig. 2, we treated the tolerant HT-29 cells with CRF, then, exposed the cells to LPS; the cell extracts were analyzed by qRT-PCR and Western blotting. The results showed that the expressions of ZO-1, ZO-2, Cldn1, Cldn3 and Cldn4 were changed slightly, but did not reach the significant criteria; the expression of Cldn2 was markedly increased in HT-29 cells at both mRNA and protein levels after the exposure to both CRF and LPS (Fig. 3A–C). The expression of Cldn2 was also confirmed by immune staining on the surface of the cells (Fig. 3D–F). We then further characterized the changes of Cldn2 in the intestinal epithelial cells. Although the simultaneous exposure to CRF and LPS (LPS was added to the apical chambers) increased the expression of Cldn2 (Fig. 3), either exposure to CRF alone (the “20#” group in Fig. 4) or LPS alone (the “0” group in Fig. 4) did not increase the expression of Cldn2. The expression could be blocked by ERK1/2 antagonists (Fig. 4). It is reported that the promoter of Cldn2 has the binding sites of NF-κB [19]; we wondered if NF-κB activation was involved in CRF-induced Cldn2 expression. To this end, a batch of HT-29 cells was pretreated with NF-κB inhibitor, diethyl maleate (DEM), and then exposed to CRF and LPS. Indeed, the expression of Cldn2 was blocked (Fig. 4).

The data we have presented so far indicate that CRF induce the TLR4 expression in HT-29 cells; ligation of TLR4 by LPS activates NF-κB, the later induces Cldn2 expression. To further confirm the inference, we transfected HT-29 cells with TLR4 gene expression plasmids, which significantly increased the expression of TLR4 in the epithelial cells (Fig.S2). After exposure of the TLR4-overexpressing epithelial cells to LPS (no CRF was added), marked increase in the expression of Cldn2 was observed (Fig. 4). The finding implies that the over-expression of Cldn2 in the epithelial cells after the exposure to CRF may be an important causative factor to induce the epithelial barrier dysfunction. To test the hypothesis, we knocked down the Cldn2 gene in the epithelial cells by gene silencing (Fig.S1B); the HT-29 monolayers were then stimulated by CRF and LPS with the procedures in Fig. 2. Indeed, the barrier function was not affected (Fig. 2).

**Figure 5:**
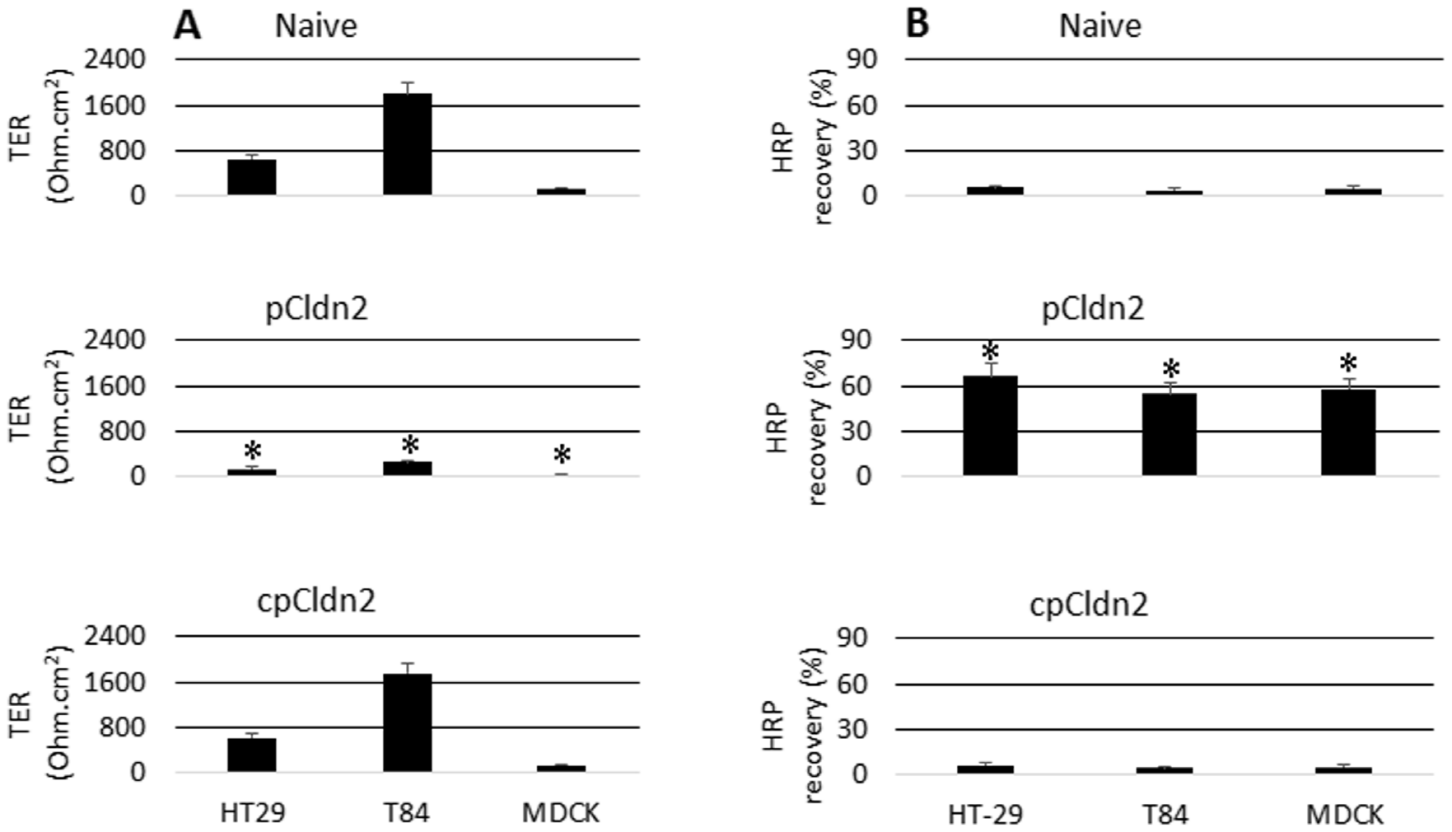
Overexpression of Cldn2 compromise epithelial monolayer barrier function. HT-29 cells and MDCK cells were transfected with pCldn2 or cpCldn2. The barrier function was evaluated. A, the bars indicate the recorded TER levels in epithelial monolayers. B, the bars indicate the contents of HRP that were recovered from the basal chambers of transwells. The data in bar graphs were presented as mean ± SD. *, p<0.01, compared with naïve groups. The data represent 5 separate experiments. pCldn2 (or cpCldn2): HT-29 cells were transfected with pCldn2 (or cpCldn2).

To further clarify the role of expression of Cldn2 in the intestinal epithelial barrier dysfunction, we constructed Cldn2-expression plasmid, pCldn2 (Fig.S2B). The epithelial cells were transfected with the pCldn2 or cpCldn2 (control). The ampicillin-resistant cells were isolated and cultured for the experiments. As detected by qRT-PCR and Western blotting, Cldn2 was expressed by the cells received the pCldn2 transfection. When cultured the Cldn2-overexpressing cells in transwells up to 4 weeks, the TER could not reach 200 Ohm/cm^2^ while the TER of naïve monolayer or cpCldn2-transfected monolayers showed increases in TER in a time-dependent manner. The permeability to macromolecular tracer, HRP, in Cldn2-overexpressing monolayer was markedly higher than the naïve or cpCldn2-transfected monolayers (Fig. 5). The results indicate that the expression of Cldn2 can compromise the epithelial barrier function.

### Psychological stress increases the expression of Cldn2 in the mouse intestinal epithelium and compromises the established endotoxin tolerance

**Figure 6:**
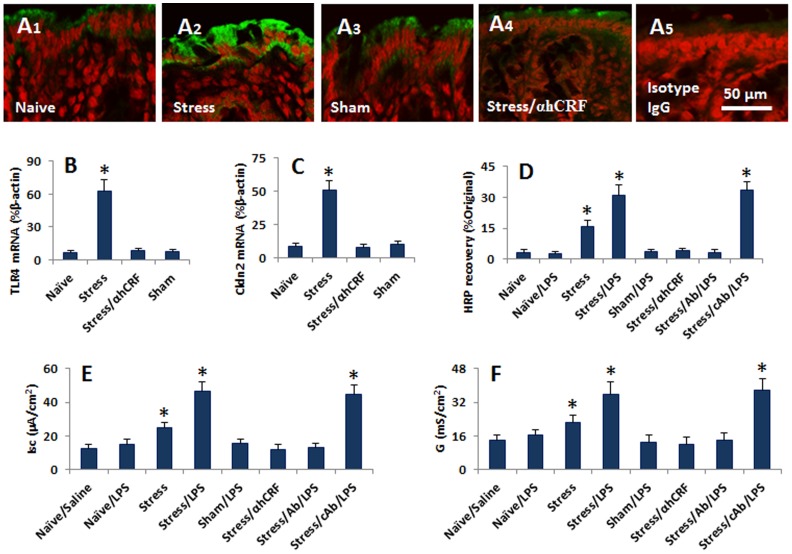
Psychological stress breaches the established epithelial endotoxin tolerance. Mice were treated with water-avoid stress daily for 10 days. The expression of Cldn2 was determined by immunohistochemistry. The colon epithelial barrier function was measured with Ussing chamber technique. A, the representative images show the expression of Cldn2 (stained in green) in colon epithelial cells. B–C, the bars indicate the mRNA levels of TLR4 (B) and Cldn2 (C) in the colon epithelium (the protein data were presented in Fig.S3). D, the bars indicate the HRP contents in the serosal side of Ussing chambers. E, the bars indicate the level of Isc of the colon epithelium. F, the bars indicate the levels of conductance (G) of the colon epithelium. Annotations: Naïve: Naïve mice. Saline: Mice were treated with saline. Stress: Mice were treated with stress. Sham: Mice were treated with sham stress. αhCRF: Mice were treated with αhCRF at a dose of 5 ng/mouse 30 min prior to each stress session. Ab (cAb): Mice were pretreated with anti-Cldn2 pAb (or isotype IgG using as a control Ab, cAb). The data in bar graphs were expressed as mean ± SD. *, p<0.01, compared with naïve or saline group. Each group consisted of 6 mice.

The data in Fig. 1–4 indicate that CRF can compromise the epithelial barrier function, which implies that psychological stress may breach the established endotoxin tolerance in the intestinal epithelium; the expression of Cldn2 may play a critical role in the process. To corroborate the findings of Fig. 1–4, we treated mice with the water-avoid stress 1 h/day for 10 days [9]. The mice were killed at the end of treatment; the colon was excised to examine the expression of Cldn2. As shown by immunohistochemistry, the positive immune products of Cldn2 were seen in the colon epithelium, in the paracellular spaces and the top of the cells (Fig. 6A). The mice treated with stress showed much stronger immune staining of Cldn2 than mice treated with sham stress. We also scratched the epithelial tissue and analyzed by qRT-PCR and ELISA for the expression of TLR4 and Cldn2. The results showed much higher expression of TLR4 and Cldn2 in stressed mice than control mice at mRNA (Fig. 6B–C) and protein levels (Fig.S3). The serum levels of corticosterone, adrenocorticotropic hormone, norepinephrine and prolactin were elevated kinetically in the period of stress (Fig.S5). The colon epithelium from naïve mice and mice treated with sham stress did not show positive response to LPS stimulation in Ussing chambers. The increases in permeability to HRP (using as a macromolecular tracer) (Fig. 6D), short circuit current (Fig. 6E) and conductance (Fig. 6F) were recorded in the intestinal epithelium of mice treated with chronic stress after stimulated by LPS in Ussing chambers, which could be abolished by pretreatment with anti-Cldn2 pAb (Fig. 6D–F). In addition, we also observed the changes in the small intestine. The data were presented in Fig.S4). The results indicate that psychological stress can breach the established epithelial endotoxin tolerance in the mouse intestine, in which CRF and Cldn2 play an important role. In addition, we also observed the increase in the expression of Cldn2 in the colon epithelial cells of patients with irritable bowel syndrome (Fig.S6).

## Discussion

The intestinal endotoxin tolerance is a unique status of the intestine to protect the intestinal tissue from the stimulation of luminal Gram negative bacteria and their products. The breakdown of the endotoxin tolerance is suggested to contribute to the pathogenesis of a number of intestinal immune disorders, such as IBD [4] and food allergy [29]; its etiology is to be further understood. The present data demonstrate that the psychological stress-derived CRF is one of the factors that can compromise the established endotoxin tolerance of the intestinal epithelium. The data indicate that the expression of TLR4 can be increased in the intestinal epithelial cells upon exposure to CRF or treatment with stress. The CRF-pulsed intestinal epithelial cells express high levels of Cldn2 after re-exposure to LPS, which results in the epithelial barrier dysfunction; the data demonstrate that the established endotoxin tolerance can be breached by CRF or psychological stress.

The phenomenon that psychological stress induces intestinal epithelial barrier dysfunction was documented by us in the previous work [7]–[11], [14] and many other investigators [30], [31]. Further studies have linked psychological stress to the pathogenesis of IBD [32] based on the facts that stress can increase the ion secretion in intestinal epithelium [7], [8], which was also recorded from the gut epithelium from IBD patients [11], [14]. The stress-induced intestinal epithelial barrier dysfunction can be abrogated by the antagonists of stress-mediator, CRF, or the receptor antagonists [7]–[9]. Expanding the above information, the present study has revealed that the stress-derived CRF increases the expression of TLR4 on intestinal epithelial cells. TLR4 recognizes the endotoxin stimulation. Ligation of TLR4 activates the target cells. The results show that upon the stimulation of CRF, the expression of TLR4 was significantly increased in HT-29 cells. The fact implicates that after stimulating by CRF or treating mice with psychological stress, the intestinal epithelial cells may over express TLR4 to gain extra capacity to respond to the stimulation of endotoxin and thus breaches the established tolerance to endotoxin. Indeed, our further experiments showed that the exposure to CRF exaggerated the response to LPS stimulation in HT-29 monolayers manifesting the drop of the TER and increase in permeability to macromolecular proteins. On the other hand, the artificial increases in TLR4 expression in HT-29 cells markedly increased the cells' response to LPS. On the other hand, the source of CRF is not only produced by the paraventricular nucleus (PVN) of the hypothalamus, some other cells, such as intestinal eosinophils [10] and cancer cells [33], also produce CRF.

Apart from increasing the production of proinflammatory cytokines by intestinal epithelial cells [3], [34], another important feature of the loss of intestinal endotoxin tolerance is the intestinal epithelial barrier dysfunction [34], through which the luminal microbial products, macromolecular antigens and other noxious substances may get the opportunity to enter the deep region of the intestine and cause further pathological responses [8], [9]. The alternation of the tight junction protein expression is one of the factors inducing epithelial barrier dysfunction. Mazzon et al observed that the reduction of zonula occludens-1 (ZO-1) was significant in the mouse intestinal epithelium after restraint stress as shown by immunohistochemistry [35]. Zhang et al found that Salmonella infection upregulates the leaky protein claudin-2 in intestinal epithelial cells [36]. Our data are somewhat in line with those previous studies [35], [36]; the contents of several tight junction proteins were also decreased, in HT-29 cells after exposure to CRF in culture, but only slightly. The difference between our data and previous reports may be because the experimental systems were different. A unique finding in our study is that CRF significantly increased the expression of Cldn2 in HT-29 cells; similar phenomenon was also observed in the mouse intestine after treated with chronic psychological stress. The finding implies that the increase in Cldn2 expression attributes the epithelial barrier dysfunction. The inference was confirmed by further experiments, in which the artificial over expression of Cldn2 resulted in HT-29 monolayer barrier dysfunction. The results are consistent with the clinical findings reported by Weber et al whom found that the expression of Cldn1 and Cldn2 was markedly increased in the intestinal epithelium of IBD patients [37]. Suzuki et al's work [19] proposes the mechanism by which the over expression of Cldn2 compromises the barrier function is that Cldn2 can form channels so as to increase the permeability of tight junctions. The initial observation about the effect of Cldn2 on tight junction function is to increase the ion permeability [19]; our data have expanded the finding by showing that the over expression of Cldn2 by epithelial cells also increase the permeability to macromolecular proteins such as HRP. Others also observed that the increase in Cldn2 even induced the bacterial translocation in the intestine [38]. On the other hand, the fact that TER was not changed after the treatment of CRF implicates that the paracellualr space may not be disturbed in the present experimental setting; the other tight junction associated proteins are less possible to be affected. The increase in the permeability of HRP after treatment with CRF may via the transcellular pathway, a phenomenon we reported previously [39].

We propose that the engagement of TLR4 increases the expression of Cldn2 in the intestinal epithelial cells. This was demonstrated by (i) blocking TLR4 abolished the CRF-induced Cldn2 expression in HT-29 cells; (ii) the artificial over expression of TLR4 increased the expression of Cldn2. Activation of NF-κB is the downstream signal transduction pathway of TLR4 activation [19]. There are NF-κB binding sites in the Cldn2 promoter sequence (−1067 to −1 bp upstream of the translational start site) [19]. Thus, it is logical that activation of TLR4 activates NF-κB, the latter induces Cldn2 promoter activation and thus increases the expression of Cldn2 in intestinal epithelial cells.

Apart from activation of TLR4, other molecules may be also involved in the mechanism of endotoxin tolerance, such as Nahid et al [40] reported that apart from TLR4, TLR2, and TLR5 were also involved in microbial product tolerance; miR146a played a critical role in the microbial product tolerance of THP-1 monocytes; knockdown of the miR146a reduced the inflammatory responses to TLR4, TLR2, and TLR5 ligands. The expression of TLR4 mainly localizes on the surface of macrophages, whereas in intestinal epithelial cells, the expression of TLR4 mainly localizes in the Golgi apparatus, which may confer different responses to LPS between macrophages and intestinal epithelial cells. In our study, we found that the stress-derived CRF up regulated the expression of TLR4 in the intestinal epithelial cells, which markedly increased the response of the intestinal epithelial cells to the endotoxin, LPS. The data are in line with others' reports. Hashimoto et al [41] indicated that prenatal stress could enhance the response to LPS in mice. Wang et al [42] also found that psychological stress increased the expression of TLR4 in cardiovascular tissue. Of course, our study may not exclude some other mechanisms by which the established endotoxin tolerance can be breached, which needs further investigation.

In summary, the exposure to CRF can induce TLR4 expression in intestinal epithelial cells; the over expression of TLR4 on intestinal epithelial cells gains the capacity to over–respond to the stimulation of LPS and induces the expression of Cldn2 in the epithelial cells; the Cldn2 compromises the intestinal epithelial barrier function and thus breaches the established intestinal endotoxin tolerance.

## Supporting Information

Figure S1 **Knockdown of genes of TLR4 and Cldn2 by gene silence.** The epithelial cells were transduced with the lentiviral vector of TLR4 shRNA or Cldn2 shRNA or control shRNA (cshRNA) respectively following the manufacturer's instruction. Cells were harvested 48 h after the transduction. The cellular protein extracts were analyzed by ELISA. The immune blots indicate the levels of TLR4 (A) and Cldn2 (B) in the cell extracts. The data were expressed as percentage of the internal control β-actin; the data represent 3 separate experiments. We also cultured the transduced cells for up to 8 weeks; the expression of TLR4 or Cldn2 was not recovered by then.
(TIF)

Figure S2 **Over expression of TLR4 and Cldn2 in epithelial cells.** The TLR4 or Cldn2 plasmids (pTLR4 or pCldn2) were purchased from Addgene (Cambridge, MA). Epithelial cells of HT-29, T84 and MDCK were transfected with pTLR4, or pCldn2, or control plasmids respectively using Lipofectamine 2000 (Invitrogen) according to the manufacturer's protocols. On the next day, the cells were treated with 50 ng/ml ampicillin and exposed to fresh media containing the same concentration of ampicillin every 3 days for 2–3 weeks. Individual drug-resistant cells were expanded for further experiments. The cell proteins were extracted from the cells and analyzed by ELISA. The bars indicate the levels of TLR4 (A) or Cldn2 (B). The data (mean ± SD) were expressed as percentage of the internal control β-actin; the data represent 3 separate experiments. Panel C shows the gene knockdown results. The data represent three separate experiments.
(TIF)

Figure S3 **The protein levels of TLR4 and Cldn2 in mouse intestinal epithelium.** Intestinal epithelial tissue was scratched from the colon of naïve mice, or mice treated with psychological stress, or stress and CRF antagonist α-helical CRF (αhCRF), or sham stress. The protein was extracted and analyzed by Western blotting. The immune blots indicate the levels of TLR4 (A) and Cldn2 (B). The integrated density of the bands were denoted above the blots. The data represent 6 separate experiments. *, p<0.01, compared with naïve group.
(TIF)

Figure S4 **Intestinal epithelial barrier permeability.** Mouse intestinal segments were mounted on Ussing chambers. FITC-dextran was added to the luminal side and sampled from the serosal side. The bars indicate the levels of dextran in the serosal side. The data were expressed as percentage of the dextran contents on the luminal side (mean ± SD). *, p<0.01, compared with the naïve/saline group. Each group consisted of 6 mice.
(TIF)

Figure S5 **Serum levels of ACTH, CORT, NE and PLC in the stressed mice.** Mice were treated with water-avoid stress as described above and sacrificed on day 0, 3, 6 and 10 respectively. The serum levels of ACTH, CORT, NE and PLC were determined by ELISA. The bars indicate the serum levels of ACTH, CORT, NE and PLC (as annotated above each panel). The data were presented as mean ± SD. *, p<0.05, compared with day 0 group. Each group consisted of 6 mice. The samples from each mouse were processed separately. The data represent six separate experiments. ACTH: Adrenocorticotropic hormone; CORT: Corticosterone; NE: Norepinephrine; PLC: Prolactin.
(TIF)

Figure S6 **Expression of Cldn2 is increased in colon mucosa of patients with irritable bowel syndrom.** Colon biopies were collected from 10 patients with irritable bowel symdrom (IBS; 5 male, age 35–60 years old; 5 female, age 32–55 years old) and 10 patients with colon polyposis (5 male, age 29–56 years old; 5 female, age 33–61 years old). The using human tissue in this study was approved by the Human Research Ethic Committee at Zhengzhou University. A informed, written consent was obtained from each subject. The biopsies were prepared for cryosections and stained by immunohistochemistry. The representative confocal images show the positive staining of Cldn2 (in green) in the colon epithelial cells.
(TIF)

Table S1 Primers using in qRT-PCR
(DOCX)

Table S2 Levels of mRNA (%β-actin) in epithelial cells after stimulated by the stress-derived molecules
(DOCX)
