# Supplementary figures and images for: Stress-Derived Corticotropin Releasing Factor Breaches Epithelial Endotoxin Tolerance
Source: PLoS One. 2013 Jun 19;8(6):e65760. doi: 10.1371/journal.pone.0065760 (PMC3686760; doi:10.1371/journal.pone.0065760)

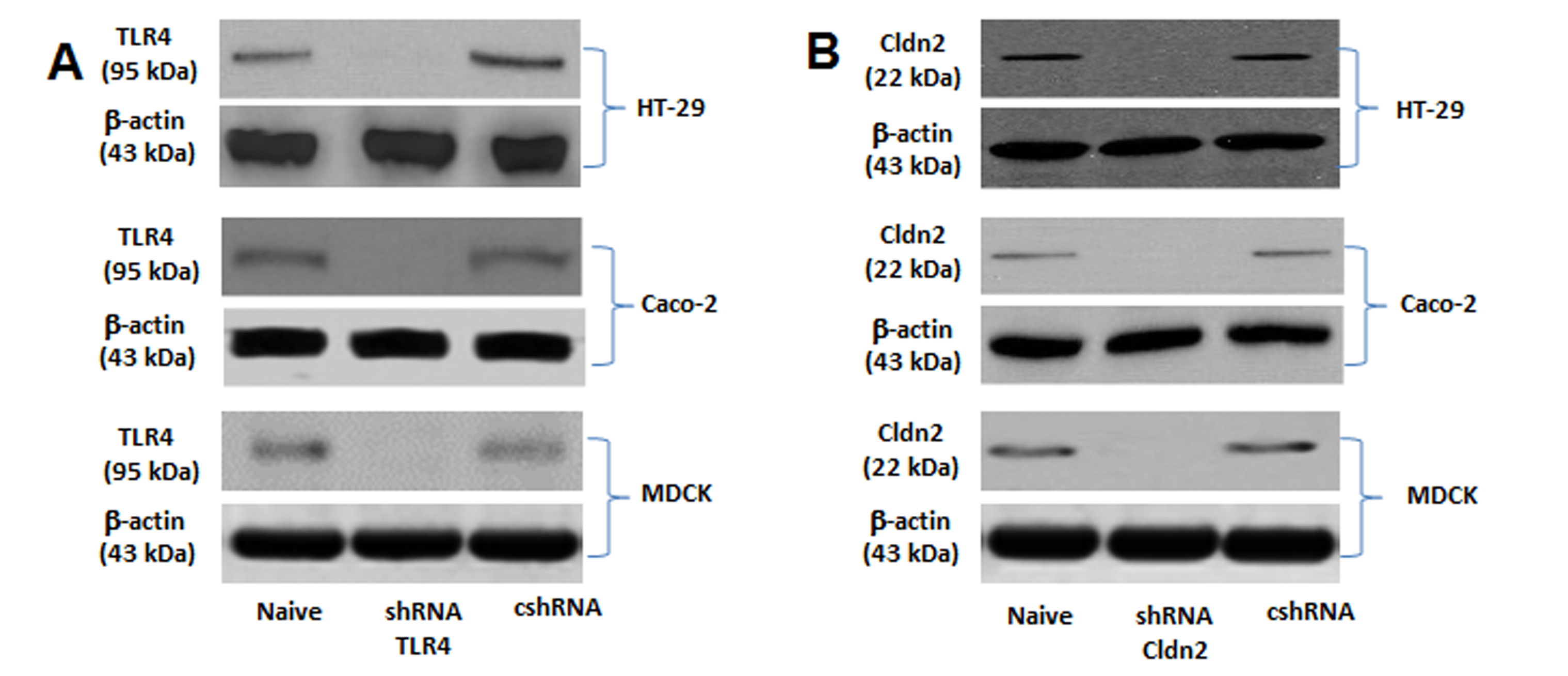

Supplement: Figure S1 — Knockdown of genes of TLR4 and Cldn2 by gene silence. The epithelial cells were transduced with the lentiviral vector of TLR4 shRNA or Cldn2 shRNA or control shRNA (cshRNA) respectively following the manufacturer's instruction. Cells were harvested 48 h after the transduction. The cellular protein extracts were analyzed by ELISA. The immune blots indicate the levels of TLR4 (A) and Cldn2 (B) in the cell extracts. The data were expressed as percentage of the internal control β-actin; the data represent 3 separate experiments. We also cultured the transduced cells for up to 8 weeks; the expression of TLR4 or Cldn2 was not recovered by then. (TIF) [file pone.0065760.s001.tif]

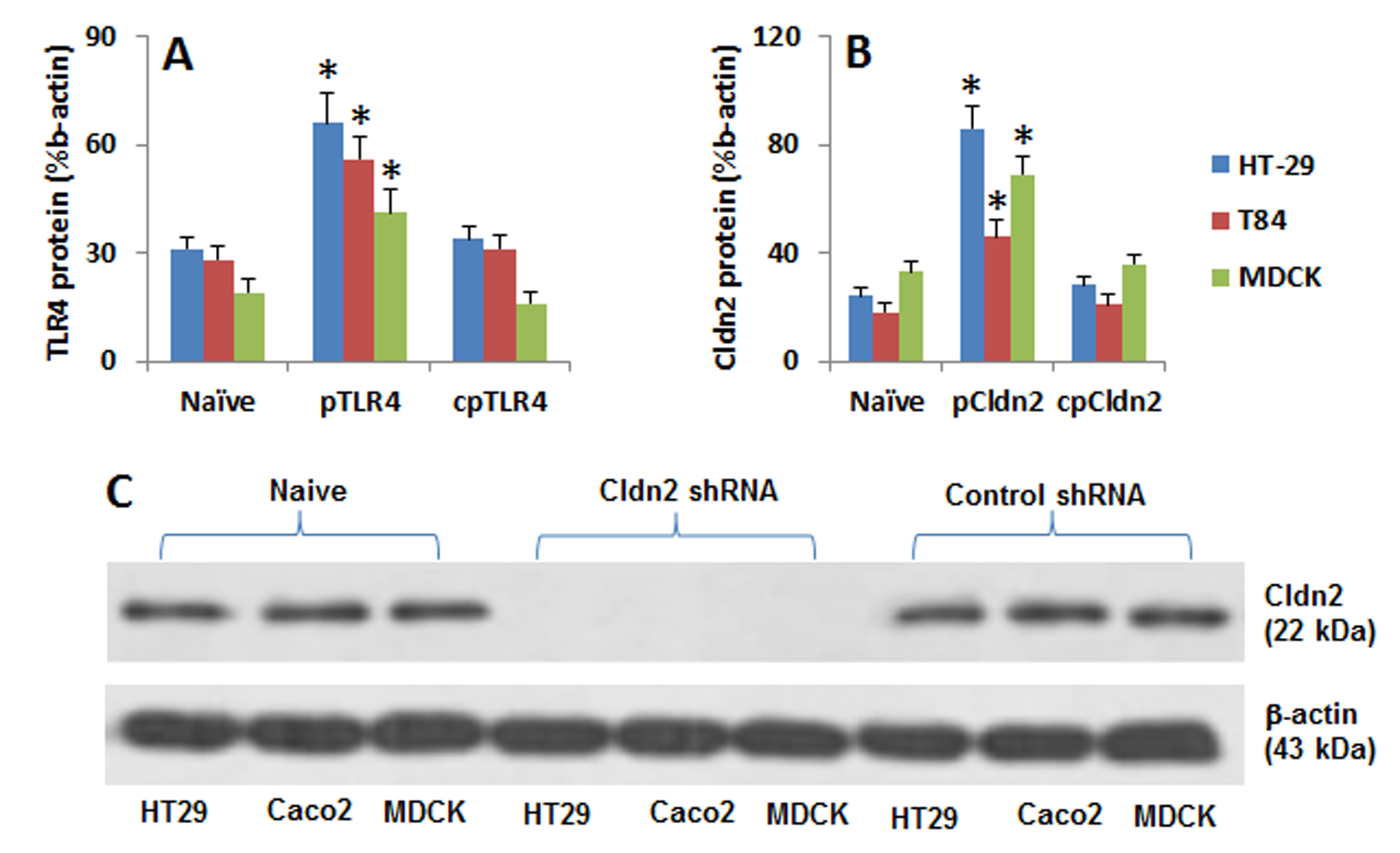

Supplement: Figure S2 — Over expression of TLR4 and Cldn2 in epithelial cells. The TLR4 or Cldn2 plasmids (pTLR4 or pCldn2) were purchased from Addgene (Cambridge, MA). Epithelial cells of HT-29, T84 and MDCK were transfected with pTLR4, or pCldn2, or control plasmids respectively using Lipofectamine 2000 (Invitrogen) according to the manufacturer's protocols. On the next day, the cells were treated with 50 ng/ml ampicillin and exposed to fresh media containing the same concentration of ampicillin every 3 days for 2–3 weeks. Individual drug-resistant cells were expanded for further experiments. The cell proteins were extracted from the cells and analyzed by ELISA. The bars indicate the levels of TLR4 (A) or Cldn2 (B). The data (mean ± SD) were expressed as percentage of the internal control β-actin; the data represent 3 separate experiments. Panel C shows the gene knockdown results. The data represent three separate experiments. (TIF) [file pone.0065760.s002.tif]

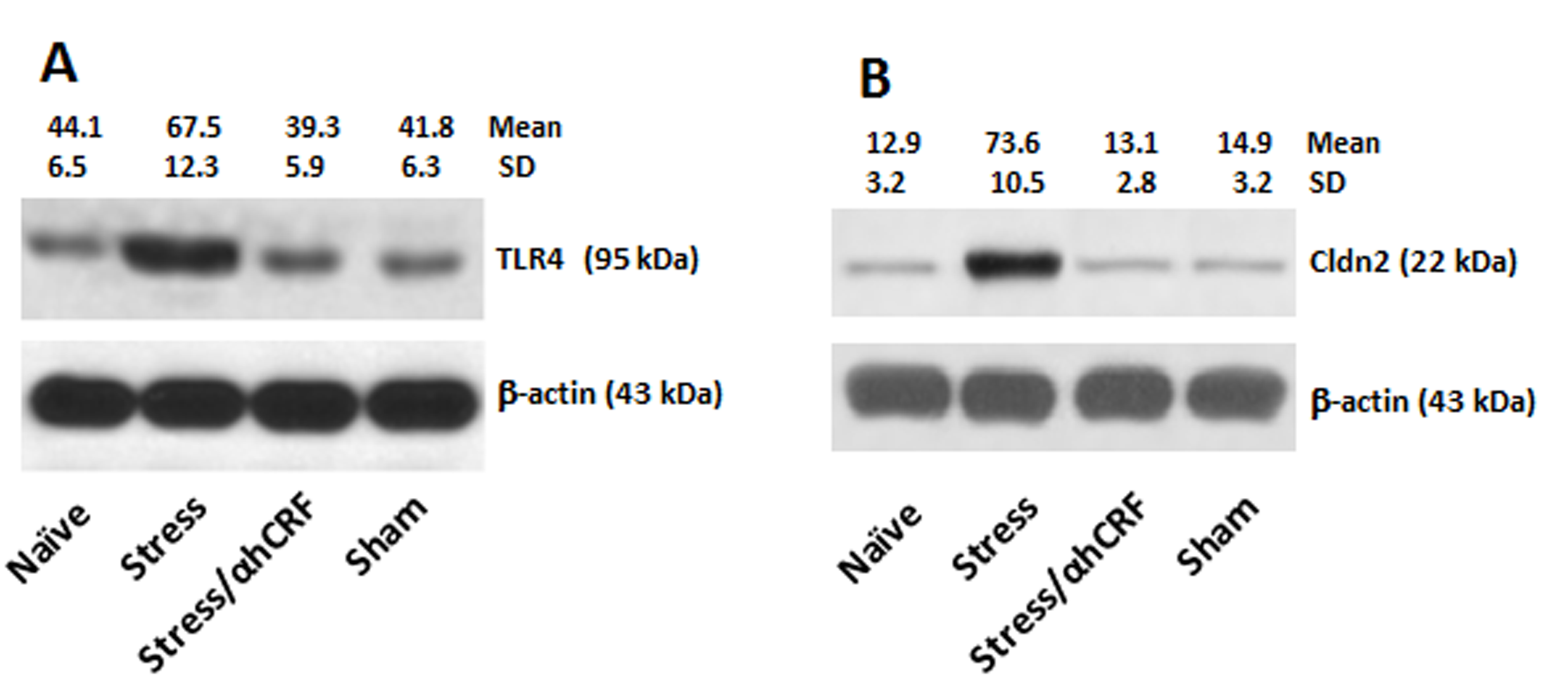

Supplement: Figure S3 — The protein levels of TLR4 and Cldn2 in mouse intestinal epithelium. Intestinal epithelial tissue was scratched from the colon of naïve mice, or mice treated with psychological stress, or stress and CRF antagonist α-helical CRF (αhCRF), or sham stress. The protein was extracted and analyzed by Western blotting. The immune blots indicate the levels of TLR4 (A) and Cldn2 (B). The integrated density of the bands were denoted above the blots. The data represent 6 separate experiments. *, p<0.01, compared with naïve group. (TIF) [file pone.0065760.s003.tif]

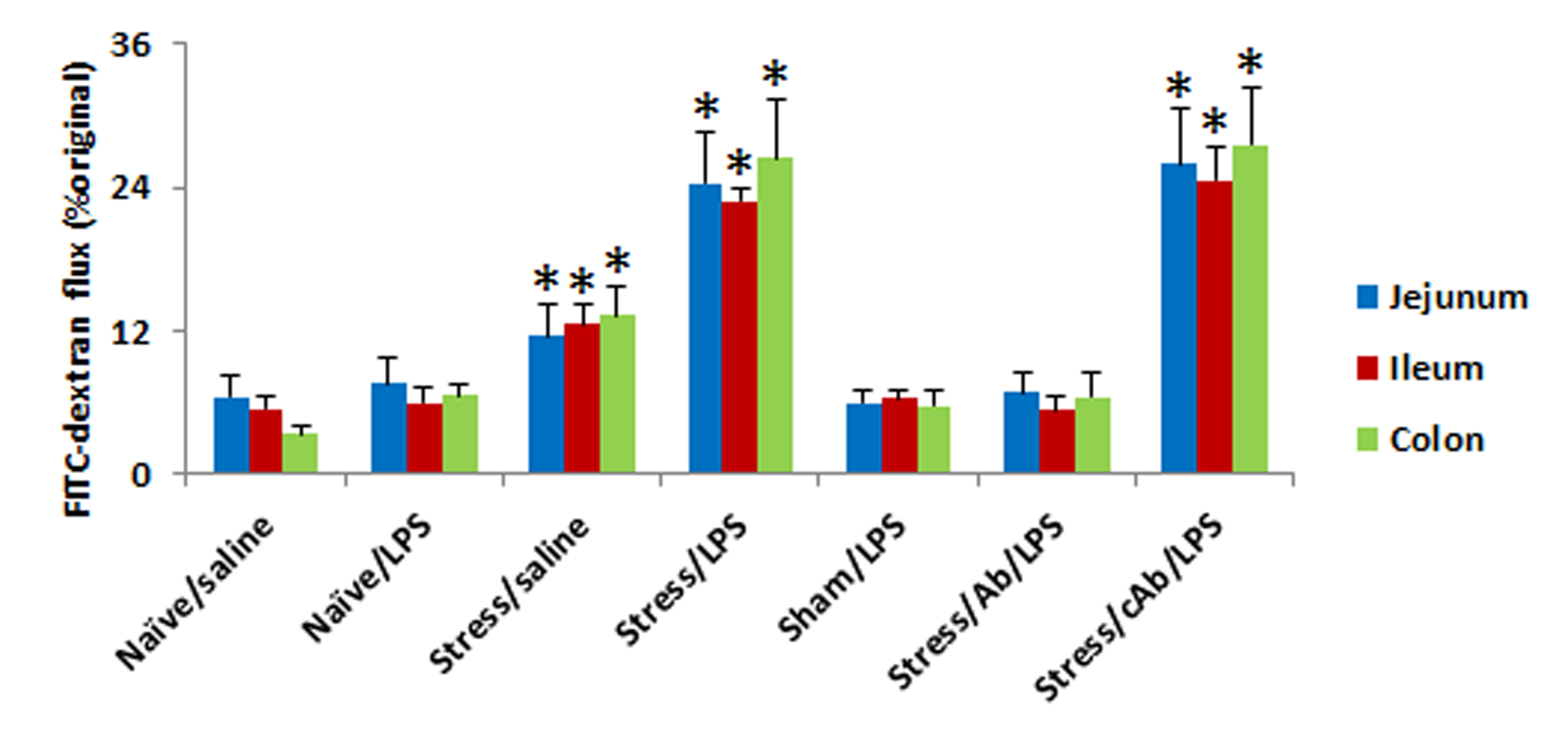

Supplement: Figure S4 — Intestinal epithelial barrier permeability. Mouse intestinal segments were mounted on Ussing chambers. FITC-dextran was added to the luminal side and sampled from the serosal side. The bars indicate the levels of dextran in the serosal side. The data were expressed as percentage of the dextran contents on the luminal side (mean ± SD). *, p<0.01, compared with the naïve/saline group. Each group consisted of 6 mice. (TIF) [file pone.0065760.s004.tif]

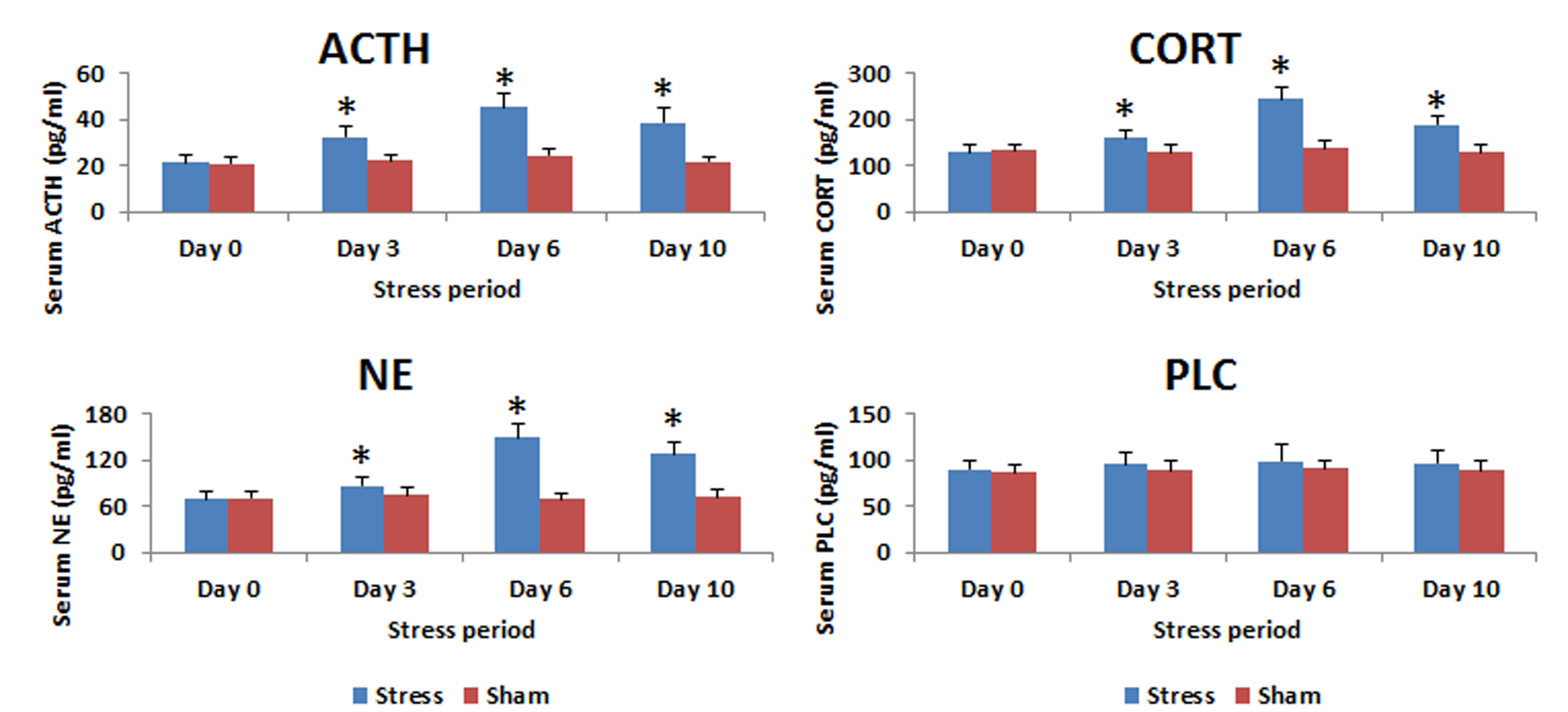

Supplement: Figure S5 — Serum levels of ACTH, CORT, NE and PLC in the stressed mice. Mice were treated with water-avoid stress as described above and sacrificed on day 0, 3, 6 and 10 respectively. The serum levels of ACTH, CORT, NE and PLC were determined by ELISA. The bars indicate the serum levels of ACTH, CORT, NE and PLC (as annotated above each panel). The data were presented as mean ± SD. *, p<0.05, compared with day 0 group. Each group consisted of 6 mice. The samples from each mouse were processed separately. The data represent six separate experiments. ACTH: Adrenocorticotropic hormone; CORT: Corticosterone; NE: Norepinephrine; PLC: Prolactin. (TIF) [file pone.0065760.s005.tif]

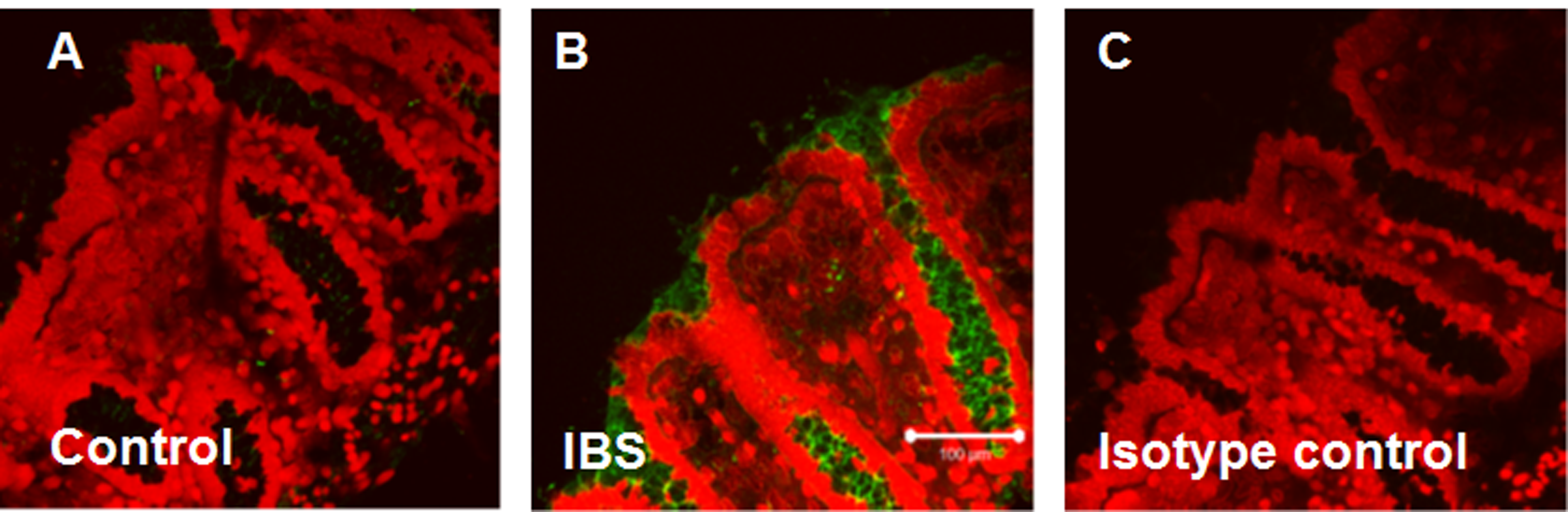

Supplement: Figure S6 — Expression of Cldn2 is increased in colon mucosa of patients with irritable bowel syndrom. Colon biopies were collected from 10 patients with irritable bowel symdrom (IBS; 5 male, age 35–60 years old; 5 female, age 32–55 years old) and 10 patients with colon polyposis (5 male, age 29–56 years old; 5 female, age 33–61 years old). The using human tissue in this study was approved by the Human Research Ethic Committee at Zhengzhou University. A informed, written consent was obtained from each subject. The biopsies were prepared for cryosections and stained by immunohistochemistry. The representative confocal images show the positive staining of Cldn2 (in green) in the colon epithelial cells. (TIF) [file pone.0065760.s006.tif]
