# Supplementary material for: Stress-Derived Corticotropin Releasing Factor Breaches Epithelial Endotoxin Tolerance
Source: PLoS One. 2013 Jun 19;8(6):e65760. doi: 10.1371/journal.pone.0065760 (PMC3686760; doi:10.1371/journal.pone.0065760)
Supplement: Table S1 — Primers using in qRT-PCR (DOCX) [file pone.0065760.s007.docx]

**Table S1. Primers using in qRT-PCR**

| Primers | Forward | Reverse | NCBI# |
| --- | --- | --- | --- |
| TLR2 (h) | gggttgaagcactggacaat | tcctgttgttggacaggtca | NM_003264.3 |
| TLR3 (h) | agccttcaacgactgatgct | tttccagagccgtgctaagt | NM_003265.2 |
| TLR4 (h) | tgagcagtcgtgctggtatc | cagggcttttctgagtcgtc | NM_138557.2 |
| TLR4 (m) | ggcagcaggtggaattgtat | aggccccagagttttgttct | NM_021297 |
| OZ-1 (h) | gaacgaggcatcatccctaa | ccagcttctcgaagaaccac | NM_175610.2 |
| OZ-2 (h) | gggatattgcaggcacagtt | cgctgtctcccttcttgaac | NM_001170414.2 |
| Cldn1 (h) | ccgttggcatgaagtgtatg | ccagtgaagagagcctgacc | BC012471 |
| Cldn2 (h) | tcgaacctcattgtcagcag | acgctgaggaagttctccaa | BC071747 |
| Cldn2 (m) | aaggtgctgctgagggtaga | ttgagcattcaaagcacagg | NM_016675.4 |
| Cldn3 (h) | aaggtgtacgactcgctgct | agtcccggataatggtgttg | AF007189 |
| Cldn4 (h) | ctccatggggctacaggtaa | agcagcgagtcgtacacctt | BC000671 |

h: Human; m: Mouse.
