# Supplementary material for: Stress-Derived Corticotropin Releasing Factor Breaches Epithelial Endotoxin Tolerance
Source: PLoS One. 2013 Jun 19;8(6):e65760. doi: 10.1371/journal.pone.0065760 (PMC3686760; doi:10.1371/journal.pone.0065760)
Supplement: Table S2 — Levels of mRNA (%β-actin) in epithelial cells after stimulated by the stress-derived molecules (DOCX) [file pone.0065760.s008.docx]

**Table S2. Levels of mRNA (%β-actin) in epithelial cells after stimulated by the stress-derived molecules**

| **Reagents** | **Saline** | **CRF** | **ACTH** | **CORT** | **NE** | **PLC** |
| --- | --- | --- | --- | --- | --- | --- |
| **TLR2** | 5.9 ± 1.3 | 5.6 ± 1.2 | 5.7 ± 1.4 | 5.2 ± 0.8 | 6.1 ± 1.4 | 6.3 ± 1.6 |
| **TLR3** | 3.8 ± 0.8 | 4.1 ± 0.9 | 1.6 ± 0.5 | 0.9 ± 0.4 | 1.2 ± 0.4 | 1.4 ± 0.5 |
| **TLR4** | 5.6 ± 1.3 | 33.2 ± 1.4 | 6.2 ± 1.8 | 4.9 ± 0.9 | 5.5 ± 1.2 | 5.8 ± 1.5 |

CRF: Cortocotropin releasing factor; ACTH: Adrenocorticotropic hormone; CORT: Corticosterone; NE: Norepinephrine; PLC: Prolactin.
